# Antibacterial Performance of Alginic Acid Coating on Polyethylene Film

**DOI:** 10.3390/ijms150814684

**Published:** 2014-08-21

**Authors:** Elika Karbassi, Ahmad Asadinezhad, Marian Lehocký, Petr Humpolíček, Alenka Vesel, Igor Novák, Petr Sáha

**Affiliations:** 1 Department of Chemical Engineering, Isfahan University of Technology, Esfahan 84156-83111, Iran; E-Mails: e.karbassi@ce.iut.ac.ir (E.K.); asadinezhad@cc.iut.ac.ir (A.A.); 2 Centre of Polymer Systems, Tomas Bata University in Zlín, Zlín 76001, Czech Republic; E-Mails: humpolicek@uni.utb.cz (P.H.); saha@utb.cz (P.S.); 3 Department of Surface Engineering, Jožef Stefan Institute, Ljubljana 1000, Slovenia; E-Mail: alenka.vesel@ijs.si; 4 Polymer Institute, Slovak Academy of Sciences, Bratislava 84236, Slovakia; E-Mail: upolnovi@savba.sk

**Keywords:** alginic acid, polyethylene, surface modification, antibacterial activity, polysaccharide

## Abstract

Alginic acid coated polyethylene films were examined in terms of surface properties and bacteriostatic performance against two most representative bacterial strains, that is, *Escherichia coli* and *Staphylococcus aureus*. Microwave plasma treatment followed by brush formation in vapor state from three distinguished precursors (allylalcohol, allylamine, hydroxyethyl methacrylate) was carried out to deposit alginic acid on the substrate. Surface analyses via various techniques established that alginic acid was immobilized onto the surface where grafting (brush) chemistry influenced the amount of alginic acid coated. Moreover, alginic acid was found to be capable of bacterial growth inhibition which itself was significantly affected by the brush type. The polyanionic character of alginic acid as a carbohydrate polymer was assumed to play the pivotal role in antibacterial activity. The cell wall composition of two bacterial strains along with the substrates physicochemical properties accounted for different levels of bacteriostatic performance.

## 1. Introduction

The surface features of biomaterials govern the interactions with biological molecules and play a decisive role in biofunctional materials development. This is why surface engineering has been practiced over recent decades to deal with a number of medical device related challenges [1]. Among numerous surface modification techniques, plasma is an effective route to activate soft matter surfaces having distinct advantages in terms of nanoscale changes on polymer surfaces while maintaining bulk properties intact [2,3,4]. Further stable enhancement of the surface reactivity can be achieved by grafting hydrophilic monomers of a vinyl type onto the surface of different substrates leading to brush formation, so that the resultant surfaces of materials reach the desired level of chemical functionality and characteristics for intended applications [5]. A functional brush not only provides desired chemistry for immobilization of bioactive entities but also prevents such entities from surface-induced denaturation of biomolecules [6]. The grafting-from approach has become the preferred option for the synthesis of polymer brushes where better control and higher reaction rate are achieved when the brush is developed in vapor phase. Viscosity in vapor state is not a determining factor, besides; there is less contamination as well as unwanted side reactions [7].

Bacterial infections are one of the critical scourges for mankind. Antibacterial agents based on biopolymers are important alternatives to low molecular weight biocides as they are usually non-toxic and can be used as effective surface coatings which inhibit bacterial proliferation [8]. Polysaccharides are polymeric carbohydrate molecules composed of long chains of monosaccharide units which are bound together by glycoside linkages. They possess versatile structural configurations and distinguished properties from their building blocks and have been studied for various biomedical applications [9,10]. Alginic acid is a carbohydrate polymer of great potential and a naturally occurring hydrophilic colloidal polysaccharide consisting mainly of residues of d-mannuronic acid and l-glucuronic acid obtained from varied species of brown seaweed [11]. It is an effective polyanion being readily associable with many molecules through ionic interactions or covalent bonds. Although there have been published many reports on alginic acid as a stand-alone material in the literature, there are few reports devoted to the alginic acid coatings on material surfaces intended for biomedical purposes [12,13,14,15,16], of which none of them has yet drawn their attention into its potential antibacterial activity. With regards to the prominent position of polyethylenes in the medical plastics arena [17,18], and to provide new insights into the bacteriostatic potentials of alginic acid, the present novel effort is aimed at modifying low-density polyethylene (LDPE) films with an aim to impart an antibacterial property. In addition, the influence of three different graft types of allyalmine, allylalcohol, and hydroxyethyl methacrylate on surface properties and alginic acid coating quality is also explored through various surface analysis tools.

## 2. Results and Discussion

To conveniently refer to the samples throughout the paper, each one is assigned a number as follows, sample 1: (untreated/control substrate), sample 2: plasma treated substrate, sample 3: allylalcohol (AAL) grafted substrate, sample 4: allylamine (AAM) grafted substrate, sample 5: 2-hydroxyethyl methacrylate (HEMA) grafted substrate, sample 6: alginic acid (ALGA) on AAL grafted substrate, sample 7: ALGA on AAM grafted substrate, and sample 8: ALGA on HEMA grafted substrate. The contact angle values of three testing liquids on untreated and modified substrates are given in Table 1. After plasma exposure, a reduction in contact angle values of three liquids on the sample surface is observed which suggests the enhanced hydrophilicity of the modified sample. Water and ethylene glycol droplets undergo further changes in terms of the angle they maintain with the solid surface compared with methylene iodide implying that polar component plays the major role in the interaction between droplet and the surface. This should result from the introduction of oxygen-containing moieties such as carbonyl, carboxyl, peroxide, and hydroperoxide on the surface. Further hydrophilicity is observed for samples 3–5 where the lowest water and ethylene glycol contact angle is exhibited by sample 5. Coating the substrate with ALGA diminishes the hydrophilicity, yet its level remains higher than that of sample 1. Such a reduction in hydrophilicity is most noticeable for sample 6. Since ALGA is hydrophilic in nature, the ALGA coated surface should typically have significant hydrophilicity. This is not clearly evident in the case of samples 6 and 7 as compared with sample 8, which could suggest that AAL and AAM are not as efficient grafts as HEMA brush. In other words, not only is HEMA more strongly bonded onto LDPE substrate, but also it shows higher reactivity towards ALGA, which facilitates coating and enhances quality of ALGA bonding as well as quantity of ALGA deposited. 

**Table 1:** Contact angle (θ) analysis results of untreated and modified substrates using deionized water (W), ethylene glycol (E), and methylene iodide (M) as wetting agents (contact angle data precision is indicated by standard deviation preceded by mean values).

Sample No.	θ_W_ (^°^)	θ_E_ (^°^)	θ_M_ (^°^)
1	92.5 ± 6.1	64.3 ± 2.5	45.8 ± 2.3
2	51.5 ± 20.2	44.4 ± 14.8	42.9 ± 23.7
3	39.4 ± 5.5	40.6 ± 6.3	50.9 ± 6.7
4	38.8 ± 6.0	37.9 ± 2.4	50.3 ± 4.6
5	35.7 ± 4.3	36.5 ± 3.8	55.9 ± 2.6
6	76.2 ± 6.7	58.1 ± 3.2	52.6 ± 5.0
7	63.0 ± 7.3	59.3 ± 4.3	54.0 ± 5.0
8	56.0 ± 6.4	50.7 ± 4.0	63.2 ± 8.2

**Figure 1:**
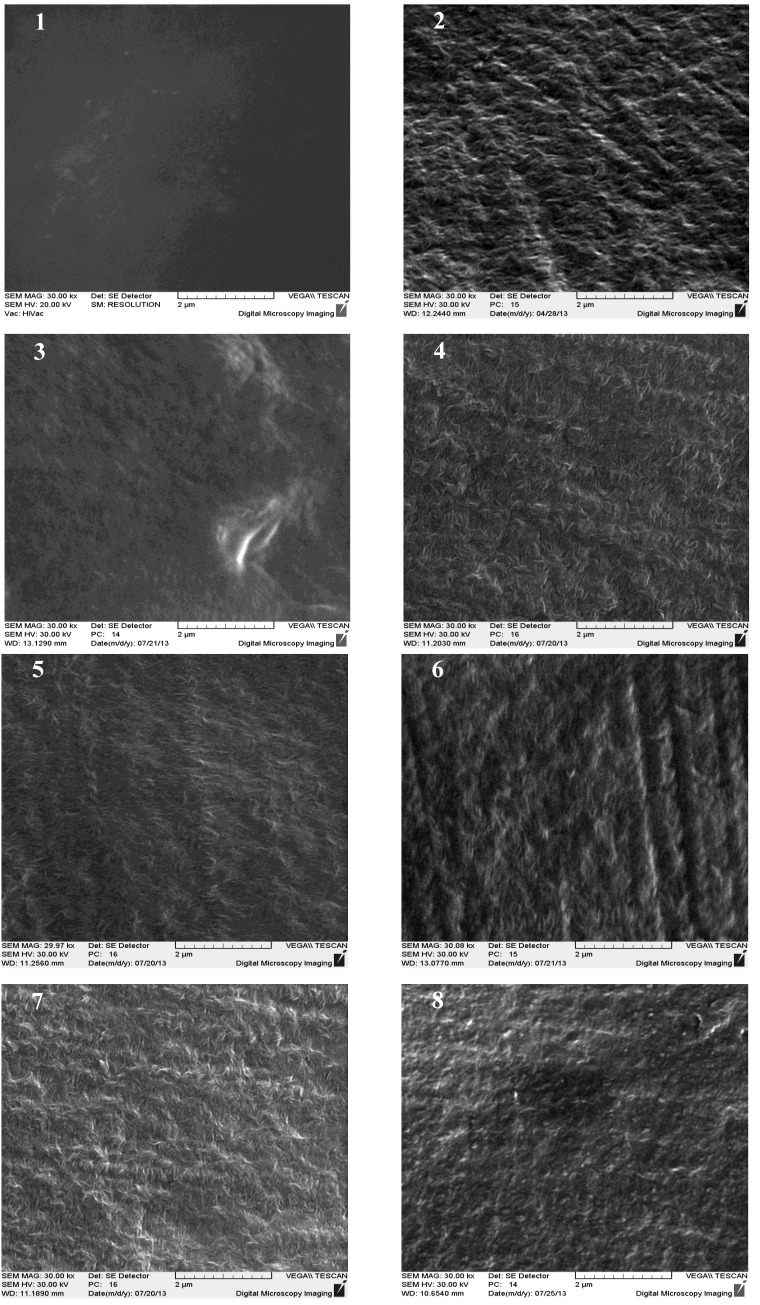
Scanning electron microscope (SEM) micrographs of untreated and modified substrates (samples 1–8) taken at 30,000× magnification.

Scanning electron microscope (SEM) images taken from untreated and modified substrates are illustrated in Figure 1. A relatively smooth, uniform morphology is well observed for sample 1 surface. Plasma treated substrate possesses quite distinct topography in terms of texture and roughness. This is normally expected after plasma in air as two simultaneous reactions (functionalization and ablation) take place as a consequence of energetic particle collision with surface molecules. Such morphology observed for sample 2 is favorable for subsequent grafting on account of increased surface area and free energy value. Upon exposing the plasma treated sample to monomer vapors (samples 3–5), a sensible change in topography comes about. Roughness slightly decreases and surface texture is significantly altered due to the strong effect of grafting on surface features. ALGA coating shows no considerable impact on surface morphology of AAL and AAM grafted substrates (samples 6 and 7) while a relatively smooth topography is seen for sample 8. Bioactive molecules are supposed to play a filling influence on surface features resulting in a comparatively level morphology. It can point to the fact that the quantity of ALGA coated is significant for sample 8 compared with samples 6 and 7. This can corroborate our contact angle analysis observation which says HEMA acts more efficiently for ALGA bonding.

**Figure 2:**
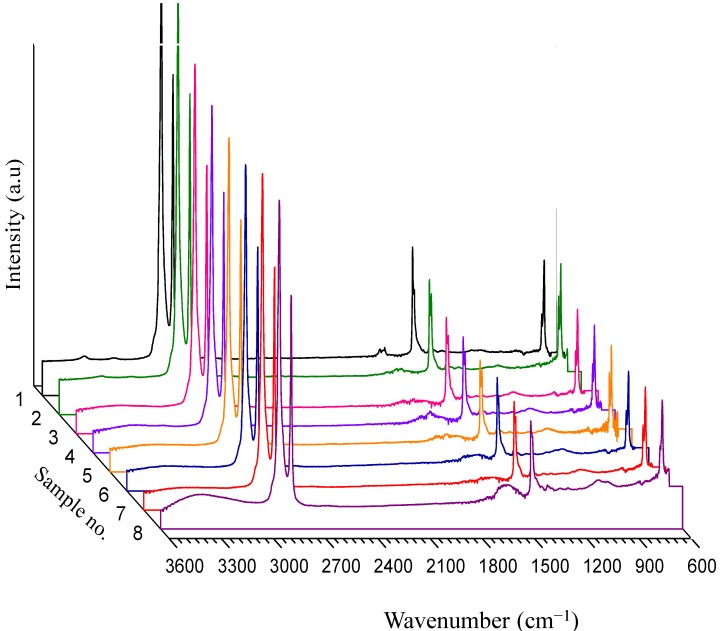
Attenuated total reflectance Fourier transform infrared (ATR-FTIR) spectra of samples 1–8 within the entire range of wavenumber.

Attenuated total reflectance Fourier transform infrared (ATR-FTIR) spectra of untreated and modified substrates in absorption mode are illustrated in Figure 2. Three characteristic sets of major peaks within 650–2950 cm^−1^ resulting from C–H bond vibrations in LDPE are easily seen, that is, one around 700 cm^−1^, the middle one at around 1500 cm^−1^ and a doublet peak around 3000 cm^−1^. They are all associated with various vibration modes of C–H bond. Due to the high probe depth of ATR-FTIR, particularly at higher wavenumbers, no detectable change in the spectrum of sample 2 is observed compared with that of sample 1 except for some slight increase in peak intensities within 700–1500 cm^−1^ which can be assigned to C–O and C–N bonds vibration. As for sample 3, the LDPE characteristic peaks intensities are reduced, besides, the signal at around 1100 cm^−1^ strengthens in magnitude which is attributed to C–O bond stretching in alcohols. Similar trends are also evident for sample 4 in which two broad peaks of minor magnitude, one at 1600 cm^−1^ and another around 1100 cm^−1^ assigned respectively to N–H bending and C–N stretching in amines are increased in magnitude. Sharper changes are evident for sample 5 where a broad peak at 1700 cm^−1^ associated with C=O group is present together with a signal around 1100 cm^−1^ due to C–O bond. Concerning samples 6 and 7, no considerable changes arise in comparison with the spectra of samples 4 and 5, however; regarding sample 8, obvious increase in C=O and C–O peaks intensity is noticeable. Moreover, a board peak within 3300–3500 cm^−1^ emerges ascribed to the hydroxyl group. The findings from FTIR analysis support observations from previous tests claiming that the HEMA graft is more favorable for ALGA coating and trace amounts of ALGA is coated on the AAL and AAM grafted samples so that they cannot be detected by ATR-FTIR probe.

Surface elemental compositions of untreated and modified substrates drawn out of the X-ray photoelectron spectroscopy (XPS) survey spectra are summarized in Table 2. Besides carbon, which is the main constituent, nitrogen and oxygen are also found in trivial amounts on sample 1, most likely due to additives or contaminants. A prominent increase in oxygen and nitrogen concentration at the expense of carbon content occurs regarding sample 2 due to the functionalization and ablation reactions after plasma exposure. Some slight changes in elemental concentrations are evident for sample 3 but not as much as expected since XPS probe depth is on the order of a few nanometers and may extend well beneath the brush. This can also indicate that the AAL layer thickness is not as high as expected most likely due to the low degree of polymerization. The same reasoning may be applied for sample 4, where an increase in nitrogen content and a reduction in oxygen concentration suggest the presence of an AAM layer on the surface, though of a very low thickness. In regard to sample 5, a reduction in nitrogen occurs as anticipated and the HEMA-grafted brush is not thick enough to extend beyond the XPS probe depth. A sharp decrease in oxygen content is observed after ALGA is coated onto the substrates (samples 6 and 7) while that of nitrogen varies depending on the graft type. Concerning sample 8, oxygen is significantly increased while nitrogen quantity remains almost constant. It is then inferred that AAL and AAM are not as efficient grafts as the HEMA graft and in other words, not only is HEMA more strongly bonded onto the LDPE substrate, but also it favors ALGA coating. This finding supports the conclusion from previous analyses. The presence of calcium and chlorine in low amount on samples 6 and 7 is most likely due to the impurities present in purchased ALGA, however the significant amount of chlorine and manganese elements on the sample 8 surface is due to potassium permanganate and hydrogen chloride used after HEMA immobilization for carboxyl group activation.

**Table 2:** Elemental composition of untreated and modified substrate surfaces calculated from respective X-ray photoelectron spectroscopy (XPS) survey spectra (experimental absolute error is ±0.5 at.%).

Sample No.	C1s (at.%)	O1s (at.%)	N1s (at.%)	Cl2p (at.%)	Mn2p (at.%)	Ca2p (at.%)
1	99.0	trace	trace	-	-	-
2	80.2	16.0	3.7	-	-	-
3	81.2	15.8	3.0	-	-	-
4	81.2	15.1	3.7	-	-	-
5	81.8	15.2	3.0	-	-	-
6	85.1	11.3	2.7	0.7	-	trace
7	85.3	11.9	1.7	0.6	-	0.5
8	68.5	20.3	2.8	5.1	1.1	2.1

**Figure 3:**
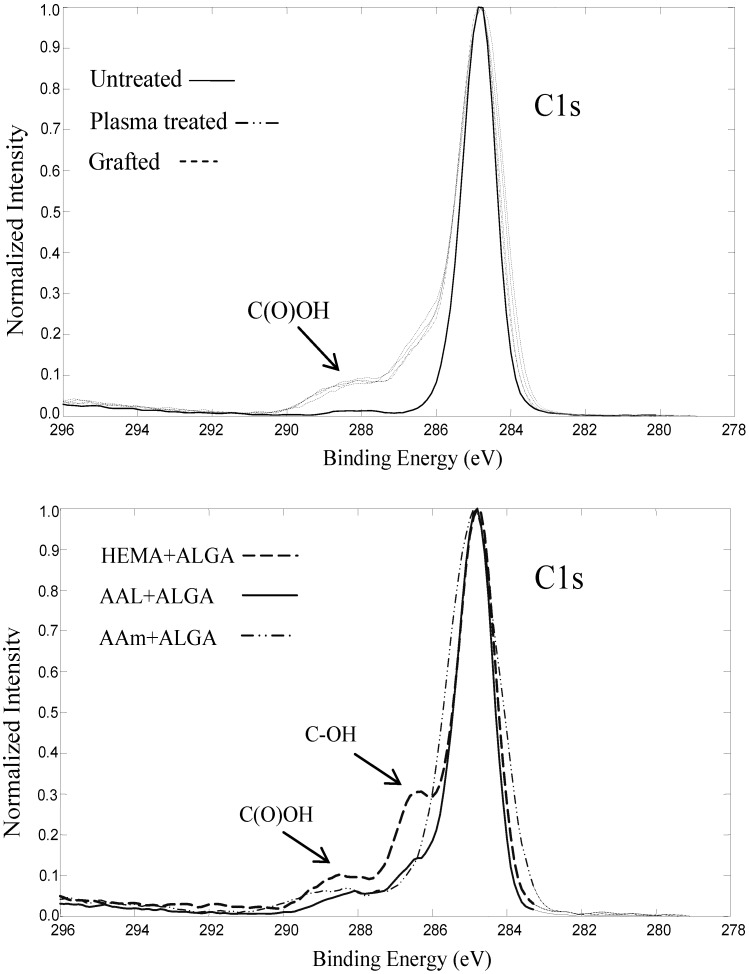
High resolution C1s peaks of samples 1–5 (**top**) and 6–8 (**bottom**) obtained from XPS analysis.

High resolution C1s signal of the samples are displayed in Figure 3. Modified substrates *versus* pristine sample have different C1s peak shapes that are directly connected with different chemical environments of the surface. A prominent peak is evident for samples 2–5 at around 289 eV which corresponds to the COOH bond. A minor shoulder adjacent to the main peak is also evident for samples 6–8 around 286.5 eV which is assigned to the COH group present in the ALGA molecule. This shoulder is meaningfully stronger for sample 8 giving credence to the aforementioned finding that HEMA acts more efficiently in coupling ALGA onto the surface.

The antibacterial capacity of the samples 1–8 based on the agar diffusion zone assay expressed as bacterial growth inhibition zone diameter are given in Table 3. Bacteriostatic agents limit the growth of bacteria by interfering with bacterial cellular metabolism, such as protein production and DNA replication. No characteristic inhibition zone is visible for samples 1–5 implying their bacteriostatic inability. As to samples 6–8, an adequate performance is evident against *E. coli* (gram negative) strain and to a lesser extent against the gram positive (*S. aureus*) strain confirming bacteriostatic capability of ALGA-coated substrates. The graft type is interestingly found to affect the performance of the samples against bacteria where the highest activity against *E. coli* and the lowest against *S. aureus* are exhibited by sample 8. The lowest activity against *E. coli* and the highest one against *S. aureus* are revealed by sample 7 and sample 6, respectively. As a matter of principle, net charge, hydrophilicity, and the amount of antibacterial agent deposited on the substrate affect the bacteriostatic quality. On the other hand, the cell wall structure and physicochemical characteristics of microorganisms are also of paramount importance. It is well recognized that *S. aureus* is different from *E. coli* in terms of cell wall composition which strongly influences the level of susceptibility. The former strain has a well-established cell wall made from a rigid peptidoglycan layer outside the cytoplasmic membrane, while the latter one possesses an outer cytoplasmic membrane made from lipopolysaccharide. Adherence of the bacteriostatic agent onto the outer cell wall and diffusivity into the membrane govern the vulnerability of the microorganism. It is therefore realized that ALGA can inhibit the bacterial growth due to its polyanionic character while coated onto a solid surface, whose level of effectiveness depends not only on the bacterial strain but also on the surface chemistry (graft type and bonding quality).

**Table 3:** Bacteriostatic performance of various substrates against two model bacteria represented by inhibition zone diameter (experimental percentage error is around ±15%).

Sample No.	1	2	3	4	5	6	7	8
*S. aureus*	n.a ^a^	n.a	n.a	n.a	n.a	14.0 mm	10.0 mm	n.a
*E. coli*	n.a	n.a	n.a	n.a	n.a	18.0 mm	16.0 mm	22.0 mm

^a^: n.a stands for “not active” which implies that the substrate showed no measurable activity.

## 3. Experimental Section

### 3.1. Materials

Low-density polyethylene film of 0.1 mm thickness was provided as packaging foil from Dow Chemicals, (Midland, MI, USA). Alginic acid from brown algae with 15%–25% carboxyl groups was provided from Sigma (St. Louis, MO, USA). Potassium permanganate, hydrochloric acid, 1-ethyl-3-(3-dimethylaminopropyl) carbodiimide, allylamine, allylalcohol, and 2-hydroxyethyl methacrylate were obtained from Aldrich (St. Louis, MO, USA). The materials were used as received without prior treatment.

### 3.2. Methods

**Figure 4:**
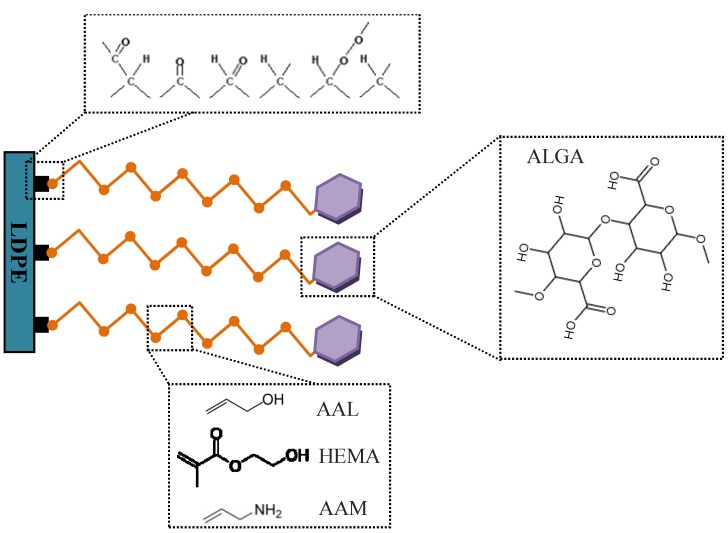
Alginic acid molecules coated onto polymeric brush spacer grown on plasma activated low-density polyethylene (LDPE) substrate.

The surface modification approach adopted in this work was founded on our previously published papers [18,19,20,21], where a multistep procedure was followed to effectively coat the substrate with bioactive molecules (Figure 4). The procedure was principally designed in such a way which could immobilize the molecules on the surface through chemical bonds. LDPE foils were first cut into 5 cm × 5 cm pieces, washed carefully with detergents to remove any surface pollutants, and fully dried in room conditions. The substrates were subjected to plasma treatment on both sides using a Pico Diener reactor (Ebhausen, Germany) operated at a pressure of 40 Pa in a microwave frequency of 2.45 GHz with power of 50 W for 60 s to generate surface reactive groups. The carrier gas was air fed to the plasma chamber at the rate of 20 standard cubic centimeters per minute. Surface-activated substrates were immediately exposed to the saturated vapor of volatile monomers of AAM, AAL, and HEMA for 10 s at room temperature for polymeric brush formation. To convert the hydroxyl groups of HEMA-exposed substrate into activated carboxyl entities, the substrate was placed in 0.05 M solution of potassium permanganate in hydrogen chloride for 1 h at 50 °C, followed by immersion in 0.1% *w*/*v* aqueous solution of carbodiimide solution for 3 h at 4 °C. Polysaccharide coating was subsequently carried out by immersing all substrates into 0.2 wt % ALGA aqueous solution of under constant shaking at 60 rpm in room temperature for 24 h. The modified substrates were finally taken out of solution, cleaned via ultrasonication with deionized water, dried, and stored in a dessicator for analysis. 

Static contact angle measurements for the surface energy estimation were performed by See System (Advex Instruments, Brno, Czech Republic) equipped with software using three standard testing liquids (water, ethylene glycol, and methylene iodide). Since water, ethylene glycol, and methylene iodide have different level of hydrophilicity, they have been employed for wettability evaluation. Water has the maximum surface tension in comparison with the other two liquids and ethylene glycol has the lowest (at room temperature). Surface tension is composed of two dispersive and polar components. Regarding water, the polar component plays the major role while in the case of ethylene glycol, the dispersive component is higher. As for methylene iodide, the polar component is zero and the whole surface tension is composed of dispersive element [22]. Sessile drop method was followed for all measurements where a 2 μL drop was laid on each substrate in room temperature and the contact angle was visualized by a camera. An average of 10 measured values was reported. Attenuated total reflectance Fourier transform infrared (ATR-FTIR) spectroscopy was performed on Thermo Scientific Avatar 320 Nicolet (Waltham, MA, USA) at spectral resolution of 2 cm^−1^ for surface chemical characterization. The spectrometer was equipped with a ZnSe crystal at an incident angle of 45°. Each spectrum represents 64 co-added scans referenced against an empty ATR cell spectrum. X-ray photoelectron spectroscopy (XPS) was conducted using XPS Physical Electronics (Minneapolis, MN, USA). The base pressure in the XPS analysis chamber was about 6 × 10^−8^ Pa. The samples were excited by X-rays over a 400 μm diameter spot area with monochromatic Al Kα1,2 radiation at 1486.6 eV. The emitted photoelectrons were detected by a hemispherical analyzer positioned at take-off angle of 45°. Survey-scan spectra were obtained at 187.85 eV pass energy and 0.4 eV step resolution. An electron gun was employed for surface neutralization. The elemental concentration analysis was performed over two different positions by the instrument software. High-resolution spectra of C1s were recorded at pass energy of 23.5 and 0.1 eV energy step. Morphological changes were tracked by SEM, VEGA II LMU (Tescan, Brno, Czech Republic) at 30,000× magnification in high vacuum/secondary electron imaging mode at an accelerating voltage of 20 kV for pristine LDPE foil and 30 kV for modified samples. The substrates were sputter coated before analysis with a thin layer of palladium/gold alloy and tilted 15° to attain qualitative insight into the level of roughness of surface features thanks to a stereoscopic view. *In vitro* antibacterial (bacteriostatic) activity was evaluated by agar diffusion method (Kirby-Bauer disk diffusion assay [23]) against two bacterial strains: *Staphylococcus aureus*, *S. aureus* 4516 (gram positive) and *Escherichia coli*, *E. coli* 4517 (gram negative) purchased from the Czech Collection of Microorganisms (Brno, Czech Republic). Circular pieces (disks) of 8 mm diameter were cut from 5 cm × 5 cm substrates, washed and dried completely, and put on nutrient agar plate M1269 from HiMedia Laboratories (Mumbai, India) inoculated with bacterial suspension (volume: 100 μL, concentration: 10^7^ units/mL). After incubation time of 24 h in 37 °C, the inhibition zone diameter was measured in five directions and the averaged value was reported. Each test was repeated in triplicate and the experimental error was calculated.

## 4. Conclusions

In this effort, low-density polyethylene films have been surface modified through microwave plasma treatment followed by grafting polymerizable monomers to provide appropriate functionalities needed for alginic acid coating. Alginic acid has been found to be effective against bacterial growth inhibition (gram positive and gram negative strains) and the level of activity depends strongly on grafting chemistry. 2-Hydroxyethyl methacrylate has been determined to be a good choice for alginic acid binding compared to allylalcohol and allyalmie, and alginic acid has been shown to be coated onto the modified surface through chemical interactions, However, only in the case of 2-hydroxyethyl methacrylate can primary covalent bonds form, and regarding allylalcohol and allylamine, secondary or ionic interactions are most likely responsible for alginic acid immobilization. The obtained findings are of value from both basic science and applied standpoints. The current effort can also underlie the forthcoming attempts to realize interactions between alginic acid and pathogenic microorganisms. From an applied viewpoint, alginic acid coatings on polymer surfaces can be useful for single-use disposable medical devices where bacterial growth inhibition is required.
